# Workplace violence against healthcare workers in the emergency department — a 10-year retrospective single-center cohort study

**DOI:** 10.1186/s13049-024-01250-w

**Published:** 2024-09-16

**Authors:** Leo Benning, Gisbert W. Teepe, Jan Kleinekort, Jorun Thoma, Michael Clemens Röttger, Andrea Prunotto, Dominik Gottlieb, Stefan Klöppel, Hans-Jörg Busch, Felix P. Hans

**Affiliations:** 1 University Emergency Center, Medical Center-University of Freiburg, Freiburg, Germany; 2 University Hospital of Old Age Psychiatry and Psychotherapy, University of Bern, Bern, Switzerland; 3 Medical Center, University of Freiburg, CNO Office, Freiburg, Germany; 4 Data Integration Center, University Medical Center Freiburg, Albert-Ludwigs-University Freiburg, Freiburg, Germany

**Keywords:** Emergency medicine, Emergency nursing, Occupational health, Personnel turnover, Aggression, Behavior and behavior mechanisms

## Abstract

**Background:**

Medical staff are regularly confronted with workplace violence (WPV), which poses a threat to the safety of both staff and patients. Structured de-escalation training (DET) for Emergency Department (ED) staff has been shown to positively affect the reporting of WPV incidents and possibly reduce its impact. This study aimed to describe the development of incidence rates, causes, means, targets, locations, responses, and the time of WPV events. Additionally, it explored the effect of the staff trained in DET on the objective and subjective severity of the respective WPV events.

**Methods:**

In a retrospective, single-center cohort study, we analyzed ten years of WPV events using the data of Staff Observation Aggression Scale-Revised (SOAS-R) score (ranging from 0 to 22) in a tertiary ED from 2014 to 2023. The events were documented by ED staff and stored in the electronic health record (EHR).

**Results:**

Between 2014 and 2023, 160 staff members recorded 859 incidents, noting an average perceived severity of 5.78 (SD = 2.65) and SOAS-R score of 11.18 (SD = 4.21). Trends showed a non-significant rise in incident rates per 10,000 patients over time. The WPV events were most frequently reported by nursing staff, and the cause of the aggression was most often not discernible (*n* = 353, 54.56%). In total, *n* = 273 (31.78%) of the WPV events were categorized as severe, and the most frequent target of the aggressive behavior was the staff. WPV events occurred most frequently in the traumatology section and the detoxification rooms. While the majority of events could be addressed with verbal interventions, more forceful interventions were performed significantly more often for higher severity WPV events. More WPV events occurred during off-hours and were of a significantly higher objective and subjective severity. Overall, the presence of staff with completed DET led to significantly higher SOAS-R scores and higher perceived severity.

**Conclusion:**

The findings underline the relevance of WPV events in the high-risk environment of an ED. The analyzed data suggest that DET significantly fostered the awareness of WPV. While most events can be addressed with verbal interventions, WPV remains a concern that needs to be addressed through organizational measures and further research.

**Supplementary Information:**

The online version contains supplementary material available at 10.1186/s13049-024-01250-w.

## Background

Violence against healthcare workers is notably prevalent in emergency department (ED) environments, with a globally increasing prevalence [1–4]. In a recent study, 73% of all nonfatal workplace injuries due to violence affected healthcare workers (HCWs) in the US [5]. Systematic reviews and meta-analysis revealed that 77% of all ED staff reported exposure to workplace violence (WPV) [6], and the pooled incidence rate of reporting was 0.0036 [7]. This fact reflects two major findings that could be identified throughout multiple healthcare systems. WPV is not a marginal phenomenon for HCWs, but rather a severe everyday problem. Affected staff may experience deleterious effects such as reduced quality of life, low self-esteem, increased anxiety, and burnout [4, 8–10] together with reduced job satisfaction, higher fluctuation of staff and — as a direct consequence — decreased patient safety and health care quality [3, 11–15].

The concept of WPV in EDs is a multifaceted phenomenon that arises from patient-related, staff-related and ED-related factors [3] and predominantly affects nursing staff [4, 6, 16]. Patients and their relatives are often under immense emotional distress due to the acute illness, injury, or altered mental state that brings the patient to the ED. A reduced emotional or cognitive capacity to respond calmly, either due to the context or due to a medical condition, can contribute to aggressive behavior towards staff members. The same applies to patients’ relatives, who are typically deeply concerned for the respective patients and overwhelmed with the situation they encounter [17–19]. Staff-related factors can be attributed to the fact that nursing staff are highly exposed and visible to instigators, becoming a frequent target for WPV [3]. ED-related factors that foster WPV may include stressful interactions between staff, patients, and visitors that are potentially aggravated by misconceptions, frustration, and anger on both sides [3, 20]. These situations might be worsened by ED events such as crowding and the absence or long waiting times for security personnel [9, 21, 22].

WPV is typically perceived as a demanding situation between the patient and the HCW, but it can also occur between nurses, physicians or other staff [23]. Here, we focused on WPV that stemmed from patients or accompanying people. Conceptually, different dimensions of aggression are distinguished in WPV. Verbal abuse is the most commonly encountered class of aggression and may include yelling, cursing, and sexual harassment [4]. Physical assaults are less common but more dramatic. Physical assaults can be performed not only with body parts such as hands or feet but also with dangerous objects such as knives, bottles, and glassware or objects from the ED infrastructure, e.g., chairs [4, 24].

HCWs themselves often consider WPV to be a routine part of their jobs and, therefore, unnecessary to report [16]. However, the careful reporting of WPV incidents provides a potential measure for understanding aggression incidents and allows the development of prevention and coping mechanisms to minimize the negative impacts of WPV [11, 16]. This lack of reporting is one of the greatest barriers to reducing workplace violence. Despite this fact, underreporting of WPV events is still a widely described phenomenon that derives from cultural, organizational, educational and behavioral aspects [2, 11, 25–29]. Contrary to this call for a better reporting culture, a recent study demonstrated that a more sophisticated reporting culture might lead to higher burnout rates due to WPV in nurses [16]. This finding might be due to the re-exposing of already negatively influenced staff to WPV contents by frequently filing these events. At the same time, an advanced reporting culture mitigates the negative effects of burnout due to WPV on patient safety [16].

The increasing awareness of the fundamental impacts of WPV has led to the development of countermeasures to foster both the incidence of WPV reporting and organizational barriers against WPV. The measures can be classified as acts of primary prevention (e.g., situational awareness, prevention programs, but also optimal staffing levels and prediction scores [30]), or secondary prevention for the immediate and effective response to violence (e.g., staff crisis intervention, reporting systems and data-driven quality management). Tertiary prevention aims to counteract the long-term negative effects of WPV [31].

De-escalation Training (DET) is designed to act as a primary, secondary, and tertiary prevention program and, therefore, is a widely accepted measure to counteract WPV and its negative consequences [32–35]. These programs offer the opportunity to acquire skill sets for early detection of potential aggression events, interact with instigators, and provide physical self-defense skills. Additionally, DET intends to improve vigilance about WPV and might foster a reporting culture. As a tertiary prevention method, DET can convey knowledge on debriefing and follow-up care for WPV victims. DET comprises teaching concepts such as hands-on training, role-play and lectures, and it can last from a few hours [32] to several days or weeks [32–34, 36, 37]. Some studies have proven that DET fosters confidence in handling WPV or the antecedent situations [32]. Other studies have shown that staff trained in de-escalation can be effectively put together in specialized teams that respond to potential WPV sites in hospitals [33, 38–42]. However, in a systematic review by Wirth et al., DET showed heterogeneous results concerning the incidence of WPV events and the reported confidence to address such events. However, Wirth et al. reported a trend toward positive effects of DET [34].

## Aim

This study first aimed to analyze the incidence rate of annual WPV-reports. Second, we aimed to investigate the causes, targets, locations, and times of the events (i.e. occurrence during on-hours or off-hours). Third, we aimed to investigate the potential effects of the DET on reporting behavior and whether the presence of de-escalation-trained staff influenced the perceived severity and the actual severity of the incidents.

## Methods

We performed a retrospective single-center cohort study in a tertiary German University ED from 2014 to 2023 to investigate the changes in reported incidences, perceived severity, and calculated severity of the incidences and the impact of trained staff present at incidents. The study used clinical routine data collected in the electronic health record (EHR), including WPV events if documented for the respective patient. We included all WPV reports in the ED-EHR without further inclusion criteria. The routine data on the count of the background population from the study site to calculate the incidence rate were extracted from the EHR accordingly. The reporting of this work follows the STROBE guidelines for observational studies [43].

## Participants and procedure

In 2014, the University Emergency Center at Freiburg University Hospital started a comprehensive prevention project. A risk analysis was conducted, and the employees were trained in de-escalation according to the ProDeMa^®^ [36] DET concept. From 2014 to 2023, 74 distinct ED staff members were trained. The aim to train all nursing staff was not achieved due to restrictions during the COVID19 pandemic. The training included instructions on the primary prevention of aggression, verbal and physical de-escalation skills, and organizational procedures for reporting cases following the procedure described below.

## Measures

The study site employed the Revised Staff Observation Aggression Scale (SOAS-R) as its primary system for documenting aggressive incidents involving patients in the EHR. The SOAS-R documentation is performed in a digital form embedded within the patient’s charts in the EHR-software and mostly consists of predefined content that the reporting staff members can check off.

Palmstierna and Wistedt initially developed the SOAS-R in 1987 [44] to record violent episodes in psychiatric settings. In 1999, the test underwent further refinement to its current iteration. The tool has been used at the study site since 2014 and is still used to report incidents by the staff. This tool is utilized by staff who observe or experience aggressive behavior from a patient. It aims to provide a structured and detailed account of the event and is separated into five distinct categories. These five categories aimed at comprehensively describing the incident are: (a) the provocation leading up to the aggression, (b) the means of aggression employed, (c) the intended goal of the aggressor, (d) the impact on the victims or object, and (e) the measures taken to mitigate the aggressive act. Each category contains multiple descriptors to describe these categories accurately [45].

To quantify the severity of an incident, the SOAS-R incorporates a scoring system in which each descriptor within the categories is assigned a point value. The score for each category is determined by the highest-scoring descriptor chosen, and the total severity score is calculated by summing the scores across all five categories. This total severity score, which can range from 0 to 22, is then classified into three severity levels: mild (0–7 points), moderate (8–15 points), and severe (16–22 points). Additionally, the SOAS-R form includes a visual analogue scale (VAS) ranging from 0 to 10, allowing staff members to assess the perceived severity of the incident. Combining the structured categorical assessment of the SOAS-R score with personal severity ratings (VAS), this dual approach enables a nuanced understanding of each aggressive event.

## Analysis

Before addressing the research questions, we reviewed the number of incidences from 2014 to 2023, the average SOAS-R score, the average perceived severity, the number of times a staff member needed to talk about the incidence and other descriptive statistics.

We used different methods to investigate the different research questions. The first question investigated the incidents’ frequency and severity change from 2014 to 2023. As our data revealed a different amount of annual patients each year to investigate the relationship between years and the number of cases, we standardized the data by calculating the yearly incidence rate (IR) as follows: IR = (Number of incidences in year / Number of patients treated in year) x Standard population (10′000).

For the incidence rate, after reviewing the data and analyzing the results from the initial linear regression, we observed a potential quadratic relationship with increasing and decreasing incidence rates. Consequently, we conducted a secondary analysis by fitting a quadratic regression model to investigate whether the incidence rate followed this quadratic pattern.

As a second and third outcome, we fitted a linear regression for the average SOAS-R score and perceived severity of that year. As a secondary confirmatory analysis, we also used a chi-square test to investigate the number of incidences for the respective assigned category (mild, moderate, severe) from 2014 to 2023.

To investigate the second research question, we reviewed the data along the different categories of the SOAS-R. We were interested in whether SOAS-R scores, perceived severity scores, and severity category differed for the interventions taken. We, therefore, used two Kruskal Wallis tests and a chi-square test. To address the difference in on-hours (08:00–17:00) and off-hours (17:00–08:00) shifts, we compared average SOAS-R scores, average perceived severity, and average number of incidences using the Mann-Whitney U test after assessing the assumptions for a parametric test. For the third question investigating the relationship between the presence of trained staff, the SOAS-R score, and perceived severity, we calculated the Pearson correlation for each pair. Finally, to approximate potential effects of the DET, we compared the average SOAS-R scores and perceived severity for incidents where no trained staff members were present versus those where at least one trained staff member was present.

As outlined in the introduction, one outcome of the DET could be to prevent, address, and mitigate incidents. To investigate this, we reviewed the correlation between the number of trained individuals, the perceived severity, and the SOAS-R score in the fourth step. In addition, we reviewed to what degree the correlation between the SOAS-R scores and the perceived severity differed when no staff with training or at least one staff member with training was present.

## Results

In total, 859 incidents were reported from 2014 to 2023 by 160 distinct ED staff members. According to the SOAS-R scores, 203 (23.63%) incidents were mild, 383 (44.59%) incidents were moderate, and 273 (31.78%) were severe. The average perceived seriousness — self-assessed scale from one to ten — was 5.78 (SD = 2.65, median = 6.00), while the mean SOAS-R — calculated from different items ranging from zero to 21 — was 11.18 (SD = 4.81). Considering that the number of trained colleagues present at the incident ranged from zero to thirteen, an average of 2.03 were present (SD = 1.52, median = 2.00). A total of 94.52% of the reports (*n* = 741) were filed by nursing staff, 3.57% by physicians (*n* = 28), 1.91% (*n* = 15) by other staff members, and 8.73% for which no data were available (*n* = 75). For 2.44% (*n* = 21) of all documented incidents, the reporting ED staff member stated a need to discuss the incident. We conducted a chi-square test to examine the association between the need for staff to talk about an incident and the severity of the incident (mild, moderate, severe). We found no significant association, *X*^2^*(2)* = 2.37, *p* = 0.31. The reports were triggered by *n* = 709 instigators; 218 (25.38%) were female, and 641 (74.62%) were male. The instigators´ age was between 18 and 96 and, on average, 45.24 (SD = 19.56) years. Ninety-five instigators caused more than one incident (13.40% of instigators, causing 28.52% of incidences; *n* = 245). Of the 95 patients with more than one incident, 18 (18.95%) were female, and 77 (81.05%) were male. The average age of these patients was 44.61 years (SD = 17.69). The highest number of reports for one patient was 14 (1.63%), a 24-year-old female patient.

### Changes in frequency, perceived severity, and SOAS-R scores

**Fig. 1:**
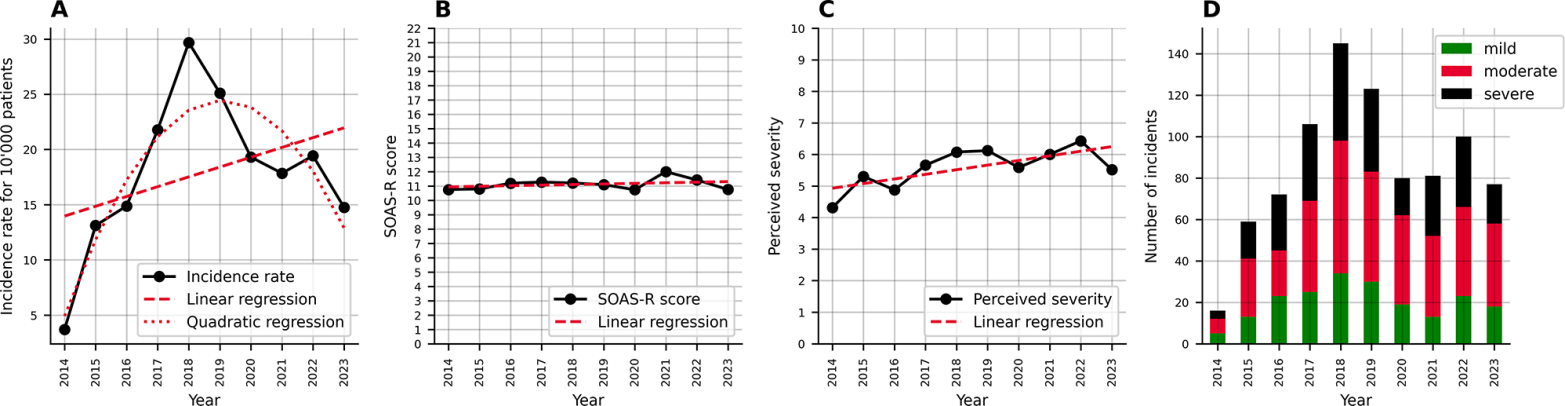
**A**: Annual incidence rate per 10,000 patients with quadratic (dotted) and linear regression (dashed). **B**: Average SOAS-R score calculated from the incidents` metrics. **C**: Average perceived severity of the incidents evaluated by the staff. **D**: Classification of the incidents (mild, moderate, severe)

Our first research question aimed to understand the change in event frequency and severity over time. To this end, we fitted three linear regressions for the incidence rate per 10’000 patients, the SOAS-R score, and the perceived severity for each year. Figure 1 illustrates the change in the number of cases (A), the change in average SOAS-R scores (B), the change in perceived severity of incidence (C) from 2014 to 2023 and the distribution of the incidents´ classification (mild, moderate, severe; Fig. 1, D).

Table 1 provides the number of cases, the average SOAS-R score, the average perceived severity of incidents for each year, and the intercept coefficient, p-value and adjusted R-squared values for each of the three linear regressions. While all three measures indicate an increase from 2014 to 2023, we found that ‘year’ did not significantly predict the change in the number of incidents and average SOAS-R scores. However, years significantly predicted an increase in the average perceived severity with a coefficient of 0.15 (*p* < 0.05), and an adjusted R-squared of 0.49. After examining the plotted incidence rate per 10’000 patients (Fig. 1, A), which indicates an increase until 2018 and a decrease after 2018, we also fitted a quadratic regression. In this quadratic regression, the model significantly predicts the dependent variable, with an adjusted R-squared value of 0.83, indicating that the model explains 83% of the variance. The linear term (coef = 7.6849, *p* < 0.01) and the quadratic term (coef = -0.7552, *p* < 0.01) were both statistically significant, suggesting a significant quadratic and linear relationship between the independent and dependent variables. Finally, we used a chi-square test to investigate whether the number of mild, moderate, or severe cases differed over the years. This test indicated that the year and the severity category were not significantly associated, *X*^*2*^ (18) = 16.22, *p* = 0.577.

**Table 1:** The table provides the incidence rates per 10’000 participants, the mean SOAS-R, the mean perceived severity for each year, and the test statistics for the linear regression

Year	Incidence Rate	Mean SOAS-*R* (SD)	Mean Perceived severity (SD)
2014	3.7	10.75 (4.95)	4.31 (2.55)
2015	13.11	10.8 (4.88)	5.31 (2.60)
2016	14.88	11.19 (5.61)	4.88 (2.83)
2017	21.79	11.27 (5.08)	5.66 (2.67)
2018	29.68	11.21 (4.7)	6.08 (2.69)
2019	25.11	11.1 (4.94)	6.12 (2.73)
2020	19.31	10.74 (4.52)	5.59 (2.43)
2021	17.85	12.0 (4.21)	6.00 (2.70)
2022	19.43	11.42 (4.73)	6.43 (2.48)
2023	14.76	10.75 (4.67)	5.52 (2.37)
**Intercept**	13.97	10.94	4.93
**Coefficient**	0.89	0.04	0.15
**p-value**	0.281	0.385	0.024
**R** ^**2**^	0.14	0.1	0.49

### Cause, means, target, location, response, and time of incidents

**Fig. 2:**
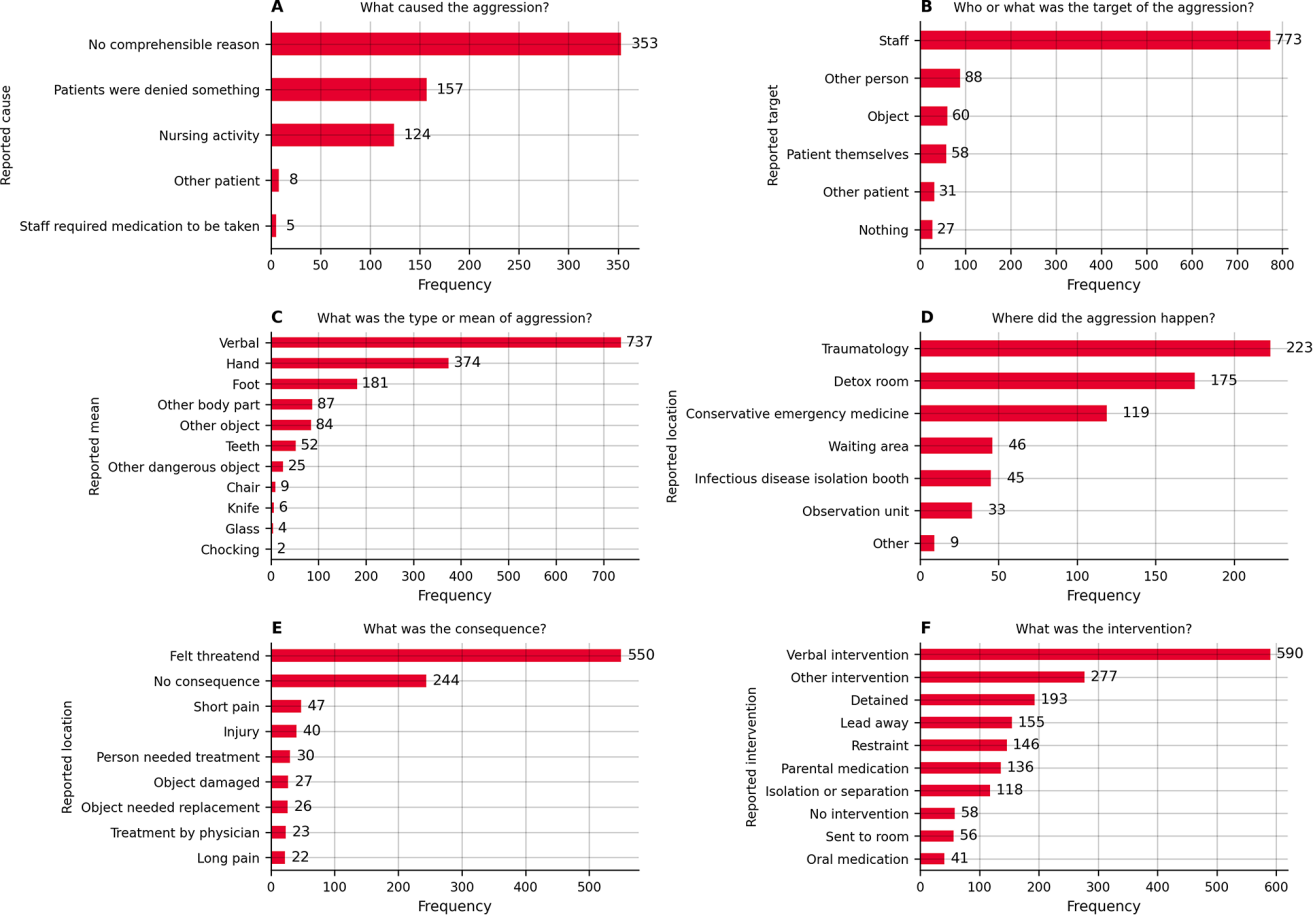
Frequencies of incidence metrics concerning the cause (**A**), the target (**B**), the type (**C**), the location (**D**), the consequences (**E**), and the taken countermeasures/intervention of the aggression (**F**). For each area (**A-F**) more than one answer could be selected. For example a aggression was caused by denying something during a nursing activity or both staff and an object were the target of the aggression

To investigate the second research question, we reviewed the incidents’ reported cause of aggression, its target, type, location, consequence, intervention, and time. Figure 2 displays the frequencies for each category, except for the time. The most common cause for aggression was not comprehensible to the documenting individual (*n* = 353, 54.56%). The second most common cause was that the patient was denied something, e.g. sedative medication, smoking, etc. (*n* = 157, 24.27%). In three-quarters of the cases, the staff was the target of the aggression (*n* = 773, 74.54%). Other people, such as police officers, were the second most frequent target of aggression (*n* = 88, 8.49%). The type of aggression was usually verbal (*n* = 737, 47.21%); however, physical aggression using hands (*n* = 374, 23.96%), feet (*n* = 181, 11.60%), and teeth (52, 3.33%) was also reported. Patients also used knives (*n* = 4, 0.26%), chairs (*n* = 9, 0.58%), glassware (*n* = 6, 0.38%), or other dangerous objects, such as shoes or infusion stands (*n* = 25, 1.6%) to threaten or hurt staff. The most common places where the aggressions occurred were the trauma treatment cubicles (*n* = 223, 25.96%), the detoxification room (*n* = 175, 20.37%), and the non-trauma cubicles (*n* = 119, 13.85%). The most reported consequence was the staff feeling threatened (*n* = 550, 54.51%). Approximately one-quarter of incidents had no consequence (*n* = 244, 24.18%). More severe consequences such as short pain (*n* = 47, 4.66%), injury (*n* = 40, 3.96%), treatment (*n* = 30, 2.97%), treatment by a physician (*n* = 26, 2.58%), and long pain (*n* = 22, 2.18%) were less frequent but had a potentially greater impact on the individual. The most common interventions or countermeasures were verbal interventions (*n* = 590, 33.33%), other interventions (*n* = 277, 15.65%), or detaining (*n* = 193, 10.9%).

**Fig. 3:**
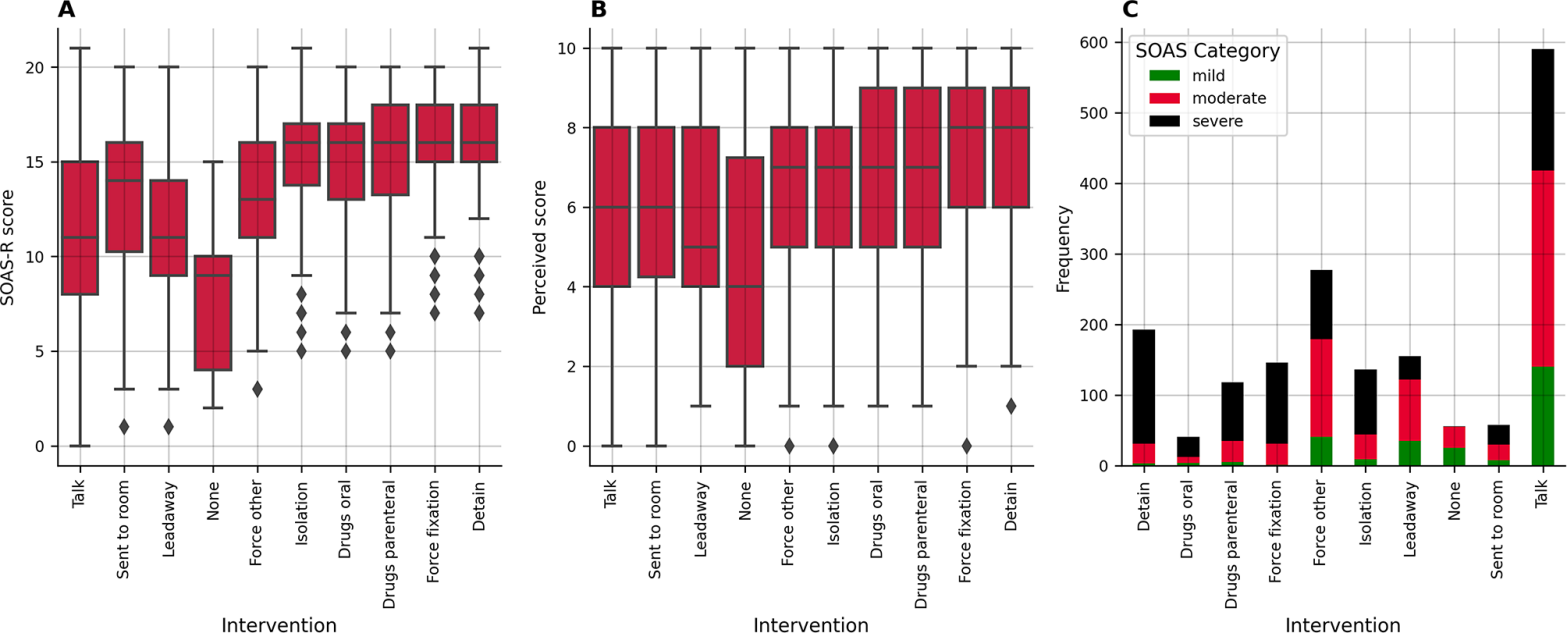
Differences based on the intervention taken for **A**: Boxplots of SOAS-R scores, **B**: Boxplots of Perceived severity, and **C**: SOAS-R category. Force other included in most cases calling the security (≈ 42%), calling the police (≈ 32%), or calling the security and the police (≈ 13%)

We investigated to what degree the SOAS-R score, perceived severity, and number of cases in each category differed for the interventions. Using a Kruskal-Wallis H test, we found that the SOAS-R scores differed for the different interventions, *H*(9) = 403.22, *p* < 0.001 (Fig. 3, A). We found the highest average SOAS-R scores when the patient was detained (M = 15.78, SD = 2.64) or forcefully fixated (M = 15.58, SD = 14.97). We observed the lowest SOAS-R score when no intervention was taken (M = 7.46, SD = 3.54). Similarly, the perceived severity also differed significantly between the undertaken interventions, H(9) = 117.12, *p* < 0.001 (Fig. 3, B). Again, we found that forcefully fixating (M = 7.33, SD = 2.17) and detaining patients (M = 7.28, SD = 2.1) had the greatest average perceived severity. We observed the lowest perceived severity, when staff performed any actions (M = 4.68, SD = 3.14). We used a chi-square test to investigate the frequency of interventions based on the different categories and found a significant impact of the category on the intervention, χ²(18) = 434.12, *p* < 0.001. For the following interventions, we found different numbers of SOAS-R-based category frequencies than we expected. Detaining an instigator was less frequent in SOAS-R-based mild events (Std Residual = -4.884) and moderate events (Std Residual = -5.411). In contrast, this intervention applied more frequently in SOAS-R-based severe events (Std Residual = 7.791), indicating a reserved application of detainment for the most severe situations. Similarly, forceful fixation of an instigator was significantly less frequent in SOAS-R-based mild events (Std Residual = -4.516) and more frequent in SOAS-R-based severe events (Std Residual = 5.854). Conversely, taking no intervention was less frequent in SOAS-R-based low events (Std Residual = 5.610) and SOAS-R-based severe events (Std Residual = -4.875). Leading the instigator away was significantly less frequently used in SOAS-R-based moderate (Std Residual = -4.527) and severe events (Std Residual = 3.474). Both results suggest that staff used these low-impact interventions only in less-treating scenarios. These patterns highlight that more severe SOAS-R categories tend to provoke more intensive interventions, reflecting a graded approach to managing varying levels of severity by the staff. This suggests that interventions are strategically escalated or de-escalated based on the assessed severity of the situation, emphasizing the adaptive nature of intervention strategies in response to the patient’s condition severity. Further descriptive statistics regarding the SOAS-R and perceived severity based on the taken interventions can be found in the Appendix Table 3, and 4.

**Fig. 4:**
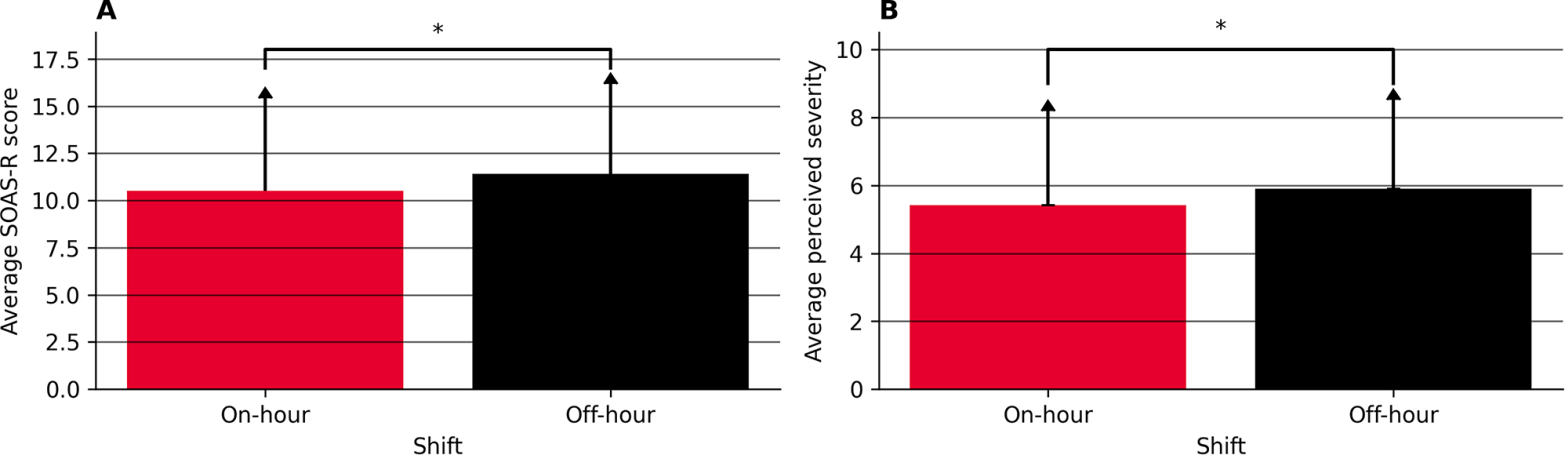
Comparison between on-hours and off-hours incidents. **A**: The difference between the SOAS-R Score. **B**: The difference between the perceived severity. The * indicates a significance with *p* < 0.05

Investigating the differences between on-hours and off-hours, we used three different outcomes, illustrated in Fig. 4. First, we compared the average SOAS-R scores for on-hours and off-hours. The Shapiro-Wilk tests indicated that the SOAR-R scores for on-hours (*W*(216) = 0.96, *p* < 0.001) and off-hours (*W*(643) = 0.96, *p* < 0.001) were not normally distributed. Given these distributions, we used a Mann-Whitney U test to compare the SOAS-R scores, indicating a significant difference between on-hours (M = 10.52, SD = 4.88) and off-hours (M = 11.40, SD = 4.77) shifts’ SOAS-R scores, *U*(857) = 62,201.50, *p* < 0.05. Plot A in Fig. 4 illustrates this difference. Second, we compared the average perceived severity for on-hours and off-hours. The Shapiro-Wilk tests indicated that the perceived severity for on-hours (*W*(216) = 0.96, *p* < 0.001) and the off-hours group (*W*(643) = 0.95, *p* < 0.001) were not normally distributed. Given these distributions, we used a Mann-Whitney U test to compare the perceived severity, indicating a significant difference in perceived severity with lower severity for on-hours (M = 5.41, SD = 2.77) than off-hours (M = 5.91, SD = 2.60), *U*(857) = 62,201.50 *p* < 0.05. Plot B in Fig. 4 illustrates this difference. These results suggest that the overall SOAS-R scores and the perceived intensity of the incidents (as reflected by the VAS score) differ significantly between shift times and a higher number of incidences during off-hours, indicating a higher perceived stress level during off-hours.

### Relationship between the number of individuals with training present, SOAS-R scores, and perceived severity

To investigate the effect of the training, we reviewed the association with the number of individuals with training present, the SOAS-R score, and the perceived severity. We used different Pearson Moment correlations analyses to investigate this relationship: the number of staff with training present significantly positively correlated with the SOAS-R scores, *r*(859) = 0.2 *p* < 0.001; the number of staff with training present and the perceived severity significantly positively correlated, *r*(859) = 0.15, *p* < 0.001; and the SOAS-R and perceived severity significantly positively correlated, *r*(859) = 0.49, *p* < 0.001. One aim was to investigate whether the training impacted the SOAS-R severity or perceived severity of the reported incidents. To this end, we used a Mann-Whitney U test to compare the average SOAS-R scores and perceived severity between incidents when staff with training were present. The average SOAS-R score was significantly higher when individuals with training were present (M = 11.4, SD = 4.81) compared to when no staff with training were present (M = 9.79, SD = 4.57), U(857) = 53772, *p* < 0.001. Similarly, the perceived severity was significantly higher when staff with training were present (M = 5.90, SD = 2.59) compared to when no staff with training were present (M = 5.07, SD = 2.90, U(857) = 52111, *p* < 0.001.

## Discussion

Compared to other professions, the risk of becoming the target of violent behavior is significantly higher for healthcare workers [46]. This circumstance has long been known [11] and initiatives to assess and counter the risks resulting from workplace violence against healthcare workers have been launched [36, 47]. Beyond these conceptual initiatives, the longitudinal coverage of events of workplace violence and its perception is important to foster the discussion on how to protect healthcare workers from such events, which is what this work contributes to the existing body of literature on WPV.

Overall, 859 incidents were filed throughout the study period, of which almost a third (31.78%) were rated severe. This finding underscores the importance of WPV in a healthcare setting that is particularly prone to violent encounters [6, 48]. In line with prior research [49], the vast majority of WPV events was experienced and documented by nursing staff (94.52%). Interestingly, the reporting staff only expressed the need to revisit and discuss the event in 2.44% of the cases. In the context of the above (i.e. high proportion of severe WPV events), it is noteworthy that no association between the severity of the WPV event and the need to revisit and discuss the event could be observed. This indicates that WPV is rarely perceived as an extraordinary event that requires some form of follow-up and is mostly considered a regular occurrence at work, which has also been described before [50, 51]. In the light of the overall trend of an increased perceived severity of the events reported, we hypothesize that understanding the reasons for a given event of aggressive behavior would help staff members to respond adequately and process the event better. This could potentially be conveyed through more structured training and regular debriefings. Yet, this hypothesis requires further research.

Revisiting our first research question, our work shows an overall increase in the incidence rates of WPV events reported (Fig. 1, A). Although the available literature does not unequivocally show increasing incidences and underscores challenges in adequate documentation of respective events, it clearly emphasizes WPV against healthcare workers as an ongoing problem [49], we deem this finding conceivable for two reasons.

Firstly, while the early years after the introduction of the SOAS-R system saw few trained staff members and low use of the newly established reporting tool, both improved over the later years after the introduction. Hence, we detected increasing incidence rates, potentially fostered by increased awareness of WPV. However, incidence rates in our study remained below what other researchers described as a pooled average for the particularly WPV-prone environment of EDs (36/10,000) [7] at all times. This indicates that the facility assessed faces similar challenges of under-reporting WPV events as previously described [52]. Specifically, other research found that only 3–23.5% of all WPV events are reported [2, 52].

Secondly, increasing patient volumes and a changing catchment population of the tertiary emergency center assessed can contribute to a changing incidence rate. Due to challenges in access to and availability of primary or specialist ambulatory care in the German healthcare system, EDs are increasingly becoming a safety net for patients seeking medical care, leading to longer waiting times and ED crowding [53, 54]. In the latter context, a positive association of high occupancy rates in the ED and the incidence rate of WPV events has been described before [55, 56].

Yet, our findings also showed a sharp and significant decline in incidence rates from 2019 (Fig. 1, A). While higher rates for WPV were documented at the time of the Covid-19-pandemic [57], poor reporting compliance as well as infrequent staff training - along with overall reduced patient volumes [58, 59] - during 2020 and 2021 can account for the relevant reduction in incidence rates. Yet, the lack of a rebound of the incidence rates in the following years remains to be investigated. While a sharp reduction in actual incidence over the duration of the pandemic is not plausible and not supported by the literature available, we assume a once again reduced awareness of the importance of the adequate reporting of WPV events. Facing record patient volumes in the center assessed, both capacity and motivation to submit additional documentation (i.e. SOAS-R scores, VAS ratings) might be reduced. While no significant increase in the SOAS-R scores could be detected over time, the perceived severity of WPV events did increase over the observation period (Table 1). The need for an increased attention to WPV events, their potential prevention and their reporting has been recognized and initiatives such as awareness campaigns have recently been launched in the ED assessed. Internationally, a zero tolerance policy towards WPV has been proposed [60], as ignoring minor WPV events is likely conducive to more serious events [61]. Furthermore, the ease of documenting WPV events is crucial and the administrative complexity of filing reports needs to be minimized. These measures, in combination with regular training on how to recognize and how to respond to WPV can help to effectively ease the strain of WPV on healthcare workers.

Our second research question aimed to assess the cause, target, means, location, response, and time of WPV incidents. Most frequently, the cause of the WPV event was indiscernible for the reporting staff (54.56%), which makes it difficult for staff to anticipate the occurrence of a potentially violent reaction (Fig. 2, A). Other causes were confrontations due to the denial of certain patient requests (e.g. smoking, sedatives; 24.27%) as well as general nursing activities (19.17%) (Fig. 2, A).

Most frequently, the WPV event was directed against staff members (75.77%) as the target of the aggression; it was directed against bystanders only rarely, respectively (Fig. 2, B). As the etiology of WPV events in healthcare settings is complex [62], the latter finding underscores the importance of staff-related safety measures, such as the avoidance of under-staffing and the improvement of training and coping/defense mechanisms [63, 64]. Environment-related safety measures include the prevention of overcrowding and long waiting times, sufficient language and culture-adapted information [63, 65].

Regarding the means of aggression, verbal aggressive behavior was reported most frequently (46.40%) (Fig. 2, C). This is well in line with the existing literature [66]. Yet, in total, all physical means of aggression accounted for the majority of the WPV events reported (use of hands: 24.17%, use of feet: 11.79%, use of teeth: 3.53%) (Fig. 2, C), which shows a discrepancy to other research [62], but underscores the relevance of providing an environment that prevents harm from staff interacting with potentially aggressive patients or visitors. Interestingly, the use of weapons (e.g. knives) or the weaponization of equipment (e.g. chairs, infusion stands) occurred only rarely while playing a more prominent role in other healthcare systems [67, 68].

WPV events occurred most frequently in the trauma treatment cubicles (28.04%) and the detoxification rooms (20.79%) (Fig. 2, D). This is a particularly plausible finding for intoxicated patients, as alcohol, prescription and illicit drugs have been identified as an important risk factors for violent behavior in EDs [69]. While no clear predisposition of traumatology patients to engage in aggressive behavior is known, this finding is plausible, as traumatology patients are often younger and male [70], these patients might have been admitted due to a physical conflict before and might concomitantly have been intoxicated with alcohol or drugs [71], which are once again known risk factors for violent behavior.

More than half of all WPV events caused the documenting staff to feel threatened (54.25%), while other consequences occurred only rarely (Fig. 2, E). 25.35% stated that no consequence resulted at all. Although the more severe consequences (e.g. pain, injuries, need to seek medical care) occur less frequently, they have a potentially more relevant impact on the reporting staff and contribute to work dissatisfaction and intention to leave their profession [66, 72]. It furthermore remains to be investigated whether the reporting of no consequences accurately reflects the perception of the WPV event, or whether this should be considered an effect of under-reporting itself. The most frequently found response to WPV events was verbal de-escalation (33.33%), which is considered the desired response, as it prevents physical harm for both staff and the instigator involved (Fig. 2, F). Yet, also more forceful responses, such as restraint, parenteral medication (i.e. sedation) and separation of the instigator were taken (Fig. 2, F). More forceful responses (i.e. forceful fixation, detention, parenteral medication) were respectively documented for more severe WPV events (Fig. 3). Further research should be conducted to evaluate whether a further increase in successful verbal de-escalations can be achieved through DET programs.

Lastly, we assessed the time of the occurrence of WPV events. Of particular interest due to its practical relevance is the distinction between shifts, often operationalized as on-hours and off-hours. Our work finds a higher number of WPV events during off-hours than during on-hours, as well as higher SOAS-R-scores and perceived severity during these times (Fig. 4, A & B). As off-hours are typically coined by the unavailability of senior staff and suboptimal outcomes for a multitude of different conditions [73], it is possible that comparable effects can also be observed for the management of aggressive behavior in an ED [48]. Yet, our work fails to establish whether this discrepancy could also be due to more resources available for additional reporting (e.g. time to submit SOAS-R scores) during off-hours and therefore leading to a detection bias.

Our third research question aimed to investigate the effect of training on the reporting of WPV events. The number of trained staff (i.e. having completed the facility’s DET, DET) being present during the WPV event led to significantly higher SOAS-R scores as well as a higher perceived severity of the events. Additionally, the SOAS-R scores and the perceived severity was higher for WPV events for which any trained staff was present. This contradicts the existing literature, which describes a reduction in perceived severity after completing designated training programs [74]. Whether our findings indicate better assessment of WPV events due to a higher awareness of the severity or whether more severe events required more experienced staff, leading to higher ratings, remains to be investigated. The beneficial effects of structured DET, however, have well been established and extend beyond the assessment of individual WPV events to improving confidence, occupational coping and self-efficacy beliefs [75].

## Limitations

This work provides a ten-year longitudinal perspective on the structured reporting of WPV events in one of Germany’s largest EDs. The selection of the study site itself might induce relevant bias, as tertiary EDs usually are located in urban areas with the respective catchment population. Therefore, our results might not reflect the conditions in smaller and rural hospitals with a lower level of care. The results warrant careful interpretation due to several other limitations. Firstly, our work is prone to detection bias, as WPV events are often under-reported. We see indicators for the same phenomenon in our work, as the incidence rate remains below what has been published as an expected average incidence rate for high-risk healthcare settings like EDs. Secondly, significant changes in the healthcare delivery environment in the area of the center assessed (stepwise implementation of an integrated emergency medicine including traumatology, internal medicine and neurology, expansion of the catchment population and increasing patient volumes, staff turnover, disruptions in patient volumes during the Covid-19 pandemic, interruptions in the DET training cadence, etc.) could potentially have introduced significant confounding into the data at hand. Thirdly, our work is based on retrospective data and can only provide directional insights and does not establish causal relationships between the factors identified and the WPV events described. Lastly, the heterogeneous use of the EHR-reporting tool might be attributed to various other reasons beyond the DET effects. The usability of digital systems might change, or the staff might be exposed to triggering events that could lead to changes in the documentation behavior. While these findings limit our work’s external validity and generalizability, they contribute to the growing body of evidence on WPV.

## Conclusion

WPV poses a significant challenge in healthcare settings in general and in the context of ED in particular. This work provides a 10-year longitudinal perspective on implementing DET for ED staff and the results thereof, collected through a WPV assessment tool in one of the largest tertiary care EDs in Germany. While lower than described in other research, our findings show an overall increase in the incidence rate of WPV events with more than 30% of all events categorized as severe. Nursing staff becomes the target of WPV over-proportionately, which most often takes the form of verbal aggression. Verbal de-escalation is the most frequent response to WPV events, and the staff only rarely reports the need to revisit and discuss the events. Yet, higher severity WPV events are associated with more forceful responses. Reports on WPV events from staff trained in DET indicate a higher severity, indicating under-reporting and underestimation of such events. These findings indicate that WPV is an ongoing concern that needs to be addressed through organizational measures and further research, but also highlights the need to improve context factors driving WPV in high-risk environments like EDs (e.g. avoidance of overcrowding and under-staffing).

## Electronic supplementary material

Below is the link to the electronic supplementary material.

Supplementary Material 1

## Data Availability

The data used for the preparation of this manuscript is available upon reasonable request, except the data concerning the reporting staff.

## Abbreviations

DET: De-escalation Training
ED: Emergency Department
EHR: Electronic Health Record
HCW: Health Care Worker
VAS: Visual Analogue Scale
WPV: Workplace Violence
