# Supplementary material for: Workplace violence against healthcare workers in the emergency department — a 10-year retrospective single-center cohort study
Source: Scand J Trauma Resusc Emerg Med. 2024 Sep 16;32:88. doi: 10.1186/s13049-024-01250-w (PMC11403778; doi:10.1186/s13049-024-01250-w)
Supplement: Supplementary file 1 — Supplementary Material 1 [file 13049_2024_1250_MOESM1_ESM.docx]

# Appendix

*Appendix* *Table 1. Descriptive statistics for (a) overall SOAS-R scores, (b) SOAS-R scores on-hours, (c) off-hours, and (d) SOAS-R scores for each year.*

|  | **N** | **Mean** | **SD** | **Min** | **Q1** | **Median** | **Q3** | **Max** |
| --- | --- | --- | --- | --- | --- | --- | --- | --- |
| Overall SOAS-R score | 725 | 10.79 | 4.93 | 0 | 7 | 11 | 15 | 21 |
| On-hours SOAS-R score | 181 | 10.6 | 4.94 | 3 | 7 | 11 | 15 | 21 |
| Off-hours SOAS-R score | 544 | 10.85 | 4.93 | 0 | 7 | 11 | 15 | 20 |
| **SOAS-R score by year** |  |  |  |  |  |  |  |  |
| 2014 | 15 | 9.93 | 5.23 | 3 | 5 | 11 | 13.5 | 18 |
| 2015 | 68 | 10.24 | 5.01 | 0 | 7 | 9 | 15 | 19 |
| 2016 | 80 | 10.8 | 5.6 | 3 | 5.75 | 11 | 16 | 19 |
| 2017 | 115 | 10.69 | 5.18 | 3 | 5.5 | 11 | 15 | 19 |
| 2018 | 155 | 10.59 | 4.79 | 3 | 7 | 11 | 15 | 20 |
| 2019 | 127 | 10.53 | 4.97 | 0 | 7 | 11 | 15 | 21 |
| 2020 | 76 | 10.38 | 4.63 | 0 | 9 | 11 | 14 | 19 |
| 2021 | 89 | 12.54 | 4.04 | 3 | 10 | 13 | 16 | 20 |

*Appendix* *Table 2. Changes in cases from 2014-2023 and the values from the fitted linear regressions.*

|  | **2014 (IR)** | **2015 (IR)** | **2016 (IR)** | **2017 (IR)** | **2018 (IR)** | **2019 (IR)** | **2020 (IR)** | **2021 (IR)** | **Δ% 2014–2021** | **Intercept** | **Slope** | ***p*** | **R2** |
| --- | --- | --- | --- | --- | --- | --- | --- | --- | --- | --- | --- | --- | --- |
| **Nursing activity** | 2 (0.46) | 4 (0.89) | 9 (1.86) | 15 (3.08) | 24 (4.91) | 12 (2.45) | 12 (2.9) | 13 (2.86) | 522 | 1.16 | 0.36 | 0.09 | 0.41 |
| **No comprehensible reason** | 5 (1.16) | 33 (7.33) | 29 (5.99) | 25 (5.14) | 60 (12.28) | 52 (10.62) | 29 (7.0) | 40 (8.81) | 659 | 4.25 | 0.87 | 0.1 | 0.38 |
| **Staff require medication intake** | 0  (0.0) | 0  (0.0) | 1 (0.21) | 2 (0.41) | 1  (0.2) | 2 (0.41) | 0  (0.0) | 0  (0.0) | - | 0.14 | 0 | 0.88 | 0 |
| **Other patient** | 0  (0.0) | 0  (0.0) | 0  (0.0) | 4 (0.82) | 0  (0.0) | 2 (0.41) | 0  (0.0) | 0  (0.0) | - | 0.14 | 0 | 0.93 | 0 |
| **Patients were denied something** | 5 (1.16) | 15 (3.33) | 25 (5.17) | 35 (7.19) | 33 (6.75) | 22 (4.49) | 13 (3.14) | 15 (3.31) | 185 | 3.83 | 0.14 | 0.69 | 0.03 |
| **Nothing or no one** | 0  (0.0) | 1 (0.22) | 0  (0.0) | 3 (0.62) | 0  (0.0) | 6 (1.23) | 3 (0.72) | 2 (0.44) | - | 0.04 | 0.1 | 0.14 | 0.33 |
| **Others (not other patients, not staff)** | 1 (0.23) | 4 (0.89) | 15 (3.1) | 12 (2.47) | 20 (4.09) | 12 (2.45) | 10 (2.41) | 6 (1.32) | 474 | 1.5 | 0.18 | 0.4 | 0.12 |
| **Other patients** | 0  (0.0) | 2 (0.44) | 1 (0.21) | 6 (1.23) | 5 (1.02) | 5 (1.02) | 1 (0.24) | 2 (0.44) | - | 0.4 | 0.05 | 0.51 | 0.08 |
| **Patient themselves** | 0  (0.0) | 5 (1.11) | 6 (1.24) | 9 (1.85) | 9 (1.84) | 7 (1.43) | 2 (0.48) | 8 (1.76) | - | 0.81 | 0.12 | 0.3 | 0.18 |
| **Staff** | 15 (3.47) | 63 (14.0) | 77 (15.91) | 101 (20.76) | 145 (29.68) | 114 (23.28) | 69 (16.65) | 81 (17.85) | 414 | 11.66 | 1.73 | 0.15 | 0.31 |
| **Objects** | 1 (0.23) | 8 (1.78) | 3 (0.62) | 11 (2.26) | 8 (1.64) | 10 (2.04) | 2 (0.48) | 7 (1.54) | 570 | 1.06 | 0.08 | 0.57 | 0.06 |
| **Feet** | 2 (0.46) | 17 (3.78) | 19 (3.93) | 28 (5.76) | 29 (5.94) | 24 (4.9) | 11 (2.65) | 27 (5.95) | 1193 | 2.68 | 0.43 | 0.16 | 0.3 |
| **Hands** | 4 (0.93) | 32 (7.11) | 40 (8.27) | 52 (10.69) | 66 (13.51) | 51 (10.41) | 29 (7.0) | 48 (10.58) | 1038 | 5.39 | 0.91 | 0.12 | 0.35 |
| **Other body parts** | 0  (0.0) | 5 (1.11) | 8 (1.65) | 14 (2.88) | 15 (3.07) | 17 (3.47) | 7 (1.69) | 10 (2.2) | - | 1.01 | 0.29 | 0.11 | 0.37 |
| **Teeth** | 0  (0.0) | 9  (2.0) | 5 (1.03) | 10 (2.06) | 8 (1.64) | 5 (1.02) | 2 (0.48) | 8 (1.76) | - | 1.07 | 0.05 | 0.69 | 0.03 |
| **Knife** | 0  (0.0) | 0  (0.0) | 1 (0.21) | 1 (0.21) | 0  (0.0) | 3 (0.61) | 0  (0.0) | 0  (0.0) | - | 0.09 | 0.01 | 0.75 | 0.02 |
| **Other dangerous objects** | 0  (0.0) | 0  (0.0) | 3 (0.62) | 4 (0.82) | 2 (0.41) | 6 (1.23) | 2 (0.48) | 2 (0.44) | - | 0.21 | 0.08 | 0.21 | 0.24 |
| **Strangulation** | 0  (0.0) | 0  (0.0) | 0  (0.0) | 1 (0.21) | 1  (0.2) | 0  (0.0) | 0  (0.0) | 1 (0.22) | - | 0.01 | 0.02 | 0.31 | 0.17 |
| **Chair** | 0  (0.0) | 0  (0.0) | 0  (0.0) | 2 (0.41) | 2 (0.41) | 2 (0.41) | 0  (0.0) | 2 (0.44) | - | 0.03 | 0.05 | 0.15 | 0.32 |
| **Glassware** | 0  (0.0) | 2 (0.44) | 0  (0.0) | 0  (0.0) | 1  (0.2) | 0  (0.0) | 0  (0.0) | 0  (0.0) | - | 0.16 | -0.02 | 0.38 | 0.13 |
| **Other common objects** | 1 (0.23) | 9  (2.0) | 9 (1.86) | 9 (1.85) | 14 (2.87) | 18 (3.68) | 4 (0.97) | 10 (2.2) | 857 | 1.33 | 0.18 | 0.3 | 0.17 |
| **Verbal aggression** | 14 (3.24) | 54 (12.0) | 65 (13.43) | 108 (22.2) | 129 (26.41) | 107 (21.85) | 66 (15.93) | 74 (16.3) | 403 | 10.56 | 1.67 | 0.14 | 0.32 |
| **No consequences** | 5 (1.16) | 25 (5.56) | 30 (6.2) | 40 (8.22) | 44 (9.01) | 38 (7.76) | 19 (4.59) | 14 (3.08) | 166 | 5.11 | 0.17 | 0.72 | 0.02 |
| **Visible injury** | 2 (0.46) | 3 (0.67) | 6 (1.24) | 9 (1.85) | 5 (1.02) | 5 (1.02) | 1 (0.24) | 5  (1.1) | 139 | 0.91 | 0.01 | 0.91 | 0 |
| **Pain for more than 10 min** | 0  (0.0) | 2 (0.44) | 1 (0.21) | 4 (0.82) | 2 (0.41) | 2 (0.41) | 1 (0.24) | 3 (0.66) | - | 0.24 | 0.05 | 0.29 | 0.18 |
| **Treatment necessary** | 0  (0.0) | 2 (0.44) | 5 (1.03) | 8 (1.64) | 3 (0.61) | 2 (0.41) | 1 (0.24) | 2 (0.44) | - | 0.64 | -0.01 | 0.91 | 0 |
| **Pain less than 10 min** | 0  (0.0) | 5 (1.11) | 9 (1.86) | 7 (1.44) | 6 (1.23) | 4 (0.82) | 2 (0.48) | 7 (1.54) | - | 0.88 | 0.05 | 0.62 | 0.04 |
| **Felt threatened** | 9 (2.08) | 35 (7.78) | 43 (8.89) | 67 (13.77) | 102 (20.88) | 82 (16.74) | 56 (13.52) | 66 (14.54) | 599 | 6.17 | 1.74 | 0.04 | 0.54 |
| **Treatment by physician necessary** | 1 (0.23) | 3 (0.67) | 3 (0.62) | 3 (0.62) | 5 (1.02) | 0  (0.0) | 1 (0.24) | 1 (0.22) | -4 | 0.61 | -0.04 | 0.44 | 0.1 |
| **Object damaged with no replacement necessary** | 0  (0.0) | 2 (0.44) | 1 (0.21) | 5 (1.03) | 7 (1.43) | 1  (0.2) | 1 (0.24) | 5  (1.1) | - | 0.29 | 0.08 | 0.34 | 0.15 |
| **Object damaged with replacement necessary** | 0  (0.0) | 2 (0.44) | 3 (0.62) | 1 (0.21) | 4 (0.82) | 6 (1.23) | 2 (0.48) | 2 (0.44) | - | 0.29 | 0.07 | 0.27 | 0.2 |
| **Forceful restraint** | 1 (0.23) | 15 (3.33) | 19 (3.93) | 28 (5.76) | 33 (6.75) | 32 (6.53) | 17 (4.1) | 24 (5.29) | 2200 | 2.49 | 0.57 | 0.07 | 0.44 |
| **Oral medication** | 2 (0.46) | 4 (0.89) | 3 (0.62) | 6 (1.23) | 4 (0.82) | 6 (1.23) | 5 (1.21) | 3 (0.66) | 43 | 0.71 | 0.05 | 0.3 | 0.18 |
| **Parenteral medication** | 0  (0.0) | 11 (2.44) | 17 (3.51) | 23 (4.73) | 29 (5.94) | 15 (3.06) | 9 (2.17) | 8 (1.76) | - | 2.5 | 0.13 | 0.68 | 0.03 |
| **Restraint** | 2 (0.46) | 11 (2.44) | 15 (3.1) | 24 (4.93) | 30 (6.14) | 22 (4.49) | 5 (1.21) | 14 (3.08) | 570 | 2.5 | 0.21 | 0.52 | 0.07 |
| **Other measures** | 7 (1.62) | 28 (6.22) | 22 (4.55) | 35 (7.19) | 43 (8.8) | 42 (8.58) | 26 (6.28) | 32 (7.05) | 335 | 4.12 | 0.62 | 0.08 | 0.43 |
| **Isolation or separation** | 1 (0.23) | 6 (1.33) | 15 (3.1) | 12 (2.47) | 15 (3.07) | 24 (4.9) | 11 (2.65) | 21 (4.63) | 1913 | 0.99 | 0.52 | 0.01 | 0.67 |
| **Calmly accompanied** | 1 (0.23) | 8 (1.78) | 9 (1.86) | 20 (4.11) | 31 (6.35) | 20 (4.08) | 19 (4.59) | 16 (3.53) | 1435 | 1.4 | 0.55 | 0.06 | 0.48 |
| **Sent to room** | 1 (0.23) | 6 (1.33) | 6 (1.24) | 5 (1.03) | 4 (0.82) | 10 (2.04) | 3 (0.72) | 6 (1.32) | 474 | 0.81 | 0.08 | 0.37 | 0.14 |
| **Verbal intervention** | 8 (1.85) | 45 (10.0) | 57 (11.78) | 64 (13.16) | 106 (21.7) | 90 (18.38) | 58 (14.0) | 65 (14.32) | 674 | 7.5 | 1.61 | 0.07 | 0.45 |

##

*Appendix Table 3: Descriptive Statistics of the SOAS-R scores based on the taken intervention.*

| **Intervention** | **N** | **mean** | **std** | **min** | **25%** | **50%** | **75%** | **max** |
| --- | --- | --- | --- | --- | --- | --- | --- | --- |
| Detain | 193 | 15.78 | 2.64 | 7 | 15 | 16 | 18 | 21 |
| Force fixation | 146 | 15.58 | 2.94 | 7 | 15 | 16 | 18 | 20 |
| Drugs parenteral | 118 | 14.97 | 3.43 | 5 | 13.25 | 16 | 18 | 20 |
| Isolation | 136 | 14.88 | 3.36 | 5 | 13.75 | 16 | 17 | 21 |
| Drugs oral | 41 | 14.68 | 3.89 | 5 | 13 | 16 | 17 | 20 |
| Sent to room | 58 | 12.98 | 4.62 | 1 | 10.25 | 14 | 16 | 20 |
| Force other | 277 | 12.59 | 3.92 | 3 | 11 | 13 | 16 | 20 |
| Talk | 590 | 10.97 | 4.78 | 0 | 8 | 11 | 15 | 21 |
| Lead away | 155 | 10.77 | 4.53 | 1 | 9 | 11 | 14 | 20 |
| None | 56 | 7.46 | 3.54 | 2 | 4 | 9 | 10 | 15 |

*Appendix Table 4: Descriptive Statistics of the perceived severity based on the taken intervention.*

| **Intervention** | **N** | **mean** | **std** | **min** | **25%** | **50%** | **75%** | **max** |
| --- | --- | --- | --- | --- | --- | --- | --- | --- |
| Force fixation | 146 | 7.33 | 2.17 | 0 | 6 | 8 | 9 | 10 |
| Detain | 193 | 7.28 | 2.1 | 1 | 6 | 8 | 9 | 10 |
| Drugs parenteral | 118 | 7.06 | 2.12 | 1 | 5 | 7 | 9 | 10 |
| Drugs oral | 41 | 6.68 | 2.67 | 1 | 5 | 7 | 9 | 10 |
| Force other | 277 | 6.56 | 2.53 | 0 | 5 | 7 | 8 | 10 |
| Isolation | 136 | 6.56 | 2.51 | 0 | 5 | 7 | 8 | 10 |
| Sent to room | 58 | 6.03 | 2.72 | 0 | 4.25 | 6 | 8 | 10 |
| Lead away | 155 | 5.83 | 2.47 | 1 | 4 | 5 | 8 | 10 |
| Talk | 590 | 5.75 | 2.54 | 0 | 4 | 6 | 8 | 10 |
| None | 56 | 4.68 | 3.14 | 0 | 2 | 4 | 7.25 | 10 |
